# Microglial responses to peripheral type 1 interferon

**DOI:** 10.1186/s12974-020-02003-z

**Published:** 2020-11-12

**Authors:** Ernest Aw, Yingying Zhang, Michael Carroll

**Affiliations:** 1 Program in Cellular and Molecular Medicine, Boston Children’s Hospital, Boston, MA USA; 2 Division of Medical Sciences, Harvard Medical School, Boston, MA USA

**Keywords:** Microglia, Interferon alpha (IFNα), Interferon-stimulated gene (ISG), Complement, Synapse engulfment, Neuropsychiatric

## Abstract

**Background:**

Interferon α (IFNα) is a cytokine whose production is increased endogenously in response to viral infection and in autoimmune diseases such as systemic lupus erythematosus (SLE). An elevated IFNα signature has been associated with clinically observed neuro-behavioural deficits such as mild cognitive impairment, fatigue, depression and psychosis in these diseases. However, the mechanisms underlying these neuropsychiatric symptoms remain largely unknown, and it is as yet unclear how IFNα signalling might influence central nervous system (CNS) function. Aberrant microglia-mediated synaptic pruning and function has recently been implicated in several neurodegenerative and neuropsychiatric diseases, but whether and how IFNα modulates these functions are not well defined.

**Methods:**

Using a model of peripheral IFNα administration, we investigated gene expression changes due to IFNAR signalling in microglia. Bulk RNA sequencing on sorted microglia from wild type and microglia-specific *Ifnar1* conditional knockout mice was performed to evaluate IFNα and IFNAR signalling-dependent changes in gene expression. Furthermore, the effects of IFNα on microglia morphology and synapse engulfment were assessed, via immunohistochemistry and flow cytometry.

**Results:**

We found that IFNα exposure through the periphery induces a unique gene signature in microglia that includes the expected upregulation of multiple interferon-stimulated genes (ISGs), as well as the complement component *C4b*. We additionally characterized several IFNα-dependent changes in microglial phenotype, including expression of CD45 and CD68, cellular morphology and presynaptic engulfment, that reveal subtle brain region-specific differences. Finally, by specifically knocking down expression of IFNAR1 on microglia, we show that these changes are largely attributable to direct IFNAR signalling on microglia and not from indirect signalling effects through other CNS parenchymal cell types which are capable of IFNα-IFNAR signal transduction.

**Conclusions:**

Peripheral IFNα induces unique genetic and phenotypic changes in microglia that are largely dependent on direct signalling through microglial IFNAR. The IFNα-induced upregulation of *C4b* could play important roles in the context of aberrant synaptic pruning in neuropsychiatric disease.

**Supplementary Information:**

**Supplementary information** accompanies this paper at 10.1186/s12974-020-02003-z.

## Introduction

Type 1 interferons are a family of cytokines classically implicated in antiviral defence, but also play important pathological roles when their expression is dysregulated, such as in the autoimmune diseases Aicardi-Goutières syndrome (AGS) and systemic lupus erythematosus (SLE) [1, 2]. Interferon α (IFNα) belongs to the type 1 interferon family, which also include IFNβ and other lesser studied IFNs; it comprises 14 different isoforms in mice and 13 in humans that are highly conserved in gene structure and amino acid sequence [3]. All type 1 interferons signal through the type 1 interferon receptors IFNAR1 and IFNAR2, which dimerize upon ligand binding and activate the JAK1 and TYK2 receptor tyrosine kinases (RTKs). Canonically, these RTKs phosphorylate the STAT1 and STAT2 transcription factors resulting in their translocation into the nucleus, where, with IRF9 they form the transcription factor complex ISGF3. ISGF3 binds to IFN-stimulated response elements (ISRE) on promoters, inducing gene expression programmes involving interferon-stimulated genes (ISGs) that are commonly antiviral and antiproliferative [3, 4]. Interestingly, the ISG expression landscape differs across cell types in a manner that is dependent on signalling strength and chronicity, as well as the activation of other non-canonical signalling machinery, resulting in functionally distinct outcomes [3, 4].

Importantly, IFNα has been closely associated with numerous neuropsychiatric comorbidities in various clinical contexts, including recombinant IFNα treatment for chronic hepatitis C infection and melanoma [5–7], as well as SLE associated neuropsychiatric disease [8, 9]. These neuropsychiatric deficiencies are heterogeneous in presentation, ranging in severity from mild cognitive dysfunction and fatigue to overt psychosis. Despite its well-studied signal transduction pathway in terms of antiviral function, it is as yet unclear how IFNα-IFNAR signalling within the brain translates to such detrimental neuropsychiatric outcomes. Furthermore, given the ubiquitous capacity of most nucleated cell types to respond to IFNAR signalling, it is unclear which cell types within the central nervous system (CNS) are critical, functional contributors to IFNα-associated neuropsychiatric disease.

Microglia are long-lived tissue-resident macrophages of the brain and play important roles in shaping neuronal circuits during development via synaptic pruning [10, 11], while also constantly surveying the brain parenchyma to detect injury or damage [12, 13]. Aberrant microglial function linked to dysregulated synaptic pruning has been implicated in the pathophysiology of several neurodegenerative and neuropsychiatric diseases including Alzheimer’s disease (AD) [14, 15], schizophrenia [16] and autism spectrum disease [17, 18]. Furthermore, microglia have been shown to secrete pro-inflammatory cytokines that negatively impact neurogenesis and influence depressive behaviour in response to peripherally derived IFNα [19], and dysregulation of IFNAR signalling in white matter microglia via genetic attenuation of its negative regulator *Usp18* causes destructive microgliopathy in the brain [20]. Interestingly, recent sequencing studies have identified a microglial type 1 IFN gene signature in the contexts of ageing [21, 22], Alzheimer’s disease [15, 23] and SLE [24], suggesting a potential unifying role of type 1 IFN signalling in propagating the neurological and neuropsychiatric pathologies in these variable pathological contexts. However, despite the increasingly appreciated role of IFNα in inflammation-associated CNS pathology, it is still unclear how IFNα regulates microglial function, and whether it might influence microglia-mediated synaptic pruning as a potential mechanism in driving IFNα-associated neuropsychiatric disease.

In the present study, we report that peripherally derived IFNα is able to transduce signalling across the blood-brain barrier (BBB), resulting in a unique microglial genetic signature that is primarily dependent on microglial IFNAR. Our results also demonstrate that in an acute model of IFNα exposure, microglia adopt a unique activated state and engulf synaptic material in a brain region-dependent manner. In light of recent studies implicating the complement system in both homeostatic and pathological synaptic pruning [10, 14, 25], the IFNα-mediated upregulation of complement component *C4b* expression in microglia may contribute to IFNα-related neuropsychiatric symptoms by a similar mechanism.

## Materials and methods

### Animals

Eight-week-old male and female C57BL/6J (000664) mice were purchased from Jackson Laboratories. Injections of PBS and murine recombinant IFNα-A (mIFNα) (BioLegend) were all performed intraperitoneally at 8 weeks of age for C57BL/6 mice. Daily injections were performed around between 0800 and 0900 h each day for 7 days, and mice were sacrificed 3 h post final injection. *Cx3cr1*-*CreERT2* (020940) and *R26*-*Eyfp*^*LSL*^ (006148) mice were purchased from Jackson Laboratories. *R26*-*Eyfp*^*LSL*^ mice were backcrossed to the C57BL/6 background in house for at least 10 generations. *Ifnar1*^*fl/fl*^ (MGI:2655303) mice were obtained from Ulrich Kalinke. *Cx3cr1*-*CreERT2* mice were crossed with *Ifnar1*^*fl/fl*^ or *R26*-*Eyfp*^*LSL*^ mice to obtain *Cx3cr1*-*CreERT2*^*+/−*^, *Ifnar*^*fl/fl*^ and *Cx3cr1*-*CreERT2*^*+/−*^, *R26*-*Eyfp*^*LSL*^ mice respectively. CreERT2 activity was induced by oral gavage of either sunflower seed oil vehicle (Spectrum Chemical) or 100 mg/kg body weight tamoxifen (Sigma) daily for 5 days at 8 weeks of age. Recycling monocytic/macrophagic populations were allowed to replenish themselves for 4 weeks [26] prior to sacrifice or mIFNα injections. Male and female mice were used for all experiments, except for the determination of serum mIFNα levels, where only male mice were used. All animal experiments were approved by the Boston Children’s Hospital and Harvard Medical School institutional animal use and care committee in accordance with NIH guidelines for the humane treatment of animals.

### Serum ELISA for mIFNα

Serum samples were assayed on the VeriPlex Mouse Cytokine 9-Plex ELISA Kit (PBL Assay Science) by contract service from the company (PBL Assay Science).

### Microglia purification

Microglia were purified using an adapted protocol [27]. Briefly, mice were perfused with ice-cold PBS and brain regions were dissected into ice-cold HBSS without calcium, magnesium and phenol red (Corning). Tissue was Dounce homogenized using a 1-mL tissue grinder (Wheaton), with 12 slow strokes each on the loose followed by the tight pestle. The cell suspension was then spun down at 1200 rpm, 7 min at 4 °C and resuspended in 70% Percoll (GE Healthcare). Thirty-seven percent Percoll was carefully layered on top and spun down at 800 g, 25 min at 23 °C with the acceleration set to 3 and deceleration set to 2 on a Sorvall Legend XTR centrifuge (Thermo Fisher). The cloudy cellular layer was then carefully pipetted and diluted in ice-cold FACS buffer (0.1% BSA, 1 mM EDTA in PBS). Cells were then processed for downstream staining for flow cytometry or fluorescence activated cell sorting (FACS). For flow cytometric assessment of presynaptic engulfment of synaptic vesicle 2 (SV2), 10 μM cytochalasin D (Sigma) was added to HBSS and all Percoll dilutions.

### Flow cytometry and sorting

Cells were stained with primary antibodies against CD11b (clone M1/70, Biolegend), CD45 (clone 30-F11, Biolegend), fixable viability dye eFluor-780 (eBioscience) for 30 min at 4 °C. For intracellular staining, cells were fixed using fixation buffer (Biolegend) and permeabilized using intracellular fixation and permeabilization buffer (Biolegend) according to manufacturer’s instructions. Fixed and permeabilized cells were stained with primary antibodies against CD68 (clone FA-11, Biolegend) and SV2 (DSHB, UIowa). SV2 monoclonal antibodies were purified and conjugated to fluorochromes in-house. Flow cytometry was performed on a FACSCanto (BD Biosciences), and microglial sorting was performed on a FACSAria II SORP (BD Biosciences) using a 70-μm nozzle. Data was analysed using FlowJo (Tree Star).

### Immunohistochemistry

Mice were perfused with ice-cold PBS, and brains were dissected and fixed in 4% PFA (Electron Microscopy Sciences) overnight at 4 °C. Tissue was cryopreserved in 30% sucrose, followed by embedding in OCT (Fisher Healthcare) and cryosectioned into 40-μm-thick floating sections. Tissue sections were blocked for 1 h at RT with blocking buffer (5% BSA, 0.2% Triton-X in PBS) and stained with primary antibody against IBA1 (Wako, 1:400) overnight at RT. Tissue sections were then washed 3× in PBS, stained with secondary antibody against rabbit IgG (Invitrogen, 1:200) for 1 h at RT, washed 3× with PBS and then mounted onto a slide with mounting medium with DAPI (Southern Biotech) and sealed with nail varnish. Primary and secondary antibodies were diluted in blocking buffer.

### Microglia morphology analysis

Confocal images of the tissue sections were obtained at × 60 magnification using the FV1000 confocal system (Olympus). Three fields of view from 2 brain sections each of the frontal cortex, hippocampus and cerebellum per biological sample were imaged and analysed using Cell Profiler [28]. Briefly, microglia were identified and segmented based on IBA1 staining, and various area and shape features were derived using the MeasureObjectSizeShape module of Cell Profiler. Pipeline used is available upon request.

### RNAscope in situ hybridization and analysis

Mouse brains were dissected and snap frozen on dry ice following terminal anaesthesia, without perfusion. OCT embedded brains were then cryosectioned into 16-μm-thick sections prior to RNAscope in situ hybridization. RNAscope in situ hybridization was performed using the RNAscope fluorescent multiplex assay kit (ACD Bio) using probes directed against *Mx1* (474931, ACD Bio), *Rsad2* (561251, ACD Bio) and *Tmem119* (472901, ACD Bio) according to manufacturer’s instructions. Confocal images were obtained at × 40 magnification using the FV1000 confocal system (Olympus). Three fields of view of the frontal cortex per biological sample were imaged and analysed using Cell Profiler [28]. Briefly, microglia were classified as DAPI+ cells containing more than 2 *Tmem119* puncta, and cells were defined as ISG positive if they expressed more than 1 identified puncta of *Mx1* or *Rsad2*. Pipeline used is available upon request.

### Gene expression by qPCR and ddPCR

Brain or spleen tissue was homogenized in TRIzol reagent (Ambion) using the TissueLyser homogenizer (Qiagen) and RNA was isolated by phenol-chloroform extraction. For microglia-specific gene expression assays, microglia were directly sorted into TCL lysis buffer (Qiagen) supplemented with 1% β-mercaptoethanol (Sigma). RNA was extracted using 2.2X RNAclean XP beads (Beckman Coulter) following manufacturer’s instructions. cDNA was then synthesized with gDNA depletion using the iScript gDNA clear cDNA synthesis kit (BioRad) following manufacturer’s instructions. qPCR reactions were assembled for genes of interest (*Actb*, *Ifnar1*) using the iTaq universal SYBR Green supermix system (BioRad) and run on a CFX96 machine (BioRad). Digital Droplet PCR (ddPCR) (BioRad) reactions were assembled according to manufacturer’s instructions, using manufacturer probes for genes of interest except for *Eif4h*, *Rpp30* and *C4b*. For qPCR, gene expression was normalized to *Actb* and compared using the *ΔΔC*_*t*_ method. For ddPCR, gene expression was normalized to *Eif4h* for microglia or *Rpp30* for spleen.

qPCR primers are as follows: *Actb* (forward: 5′ AAGAGCTATGAGCTGCCTGA reverse: 5′ TACGGATGTCAACGTCACAC) and *Ifnar1* (forward: 5′TGTGCTTCCCACCACTCAAG, reverse: 5′ AGGCGCGTGCTTTACTTCTA).

ddPCR primer/probe sets are as follows: *Mx1* (dMmuCPE5121908, BioRad), *Ifit3* (dMmuCPE5197126, BioRad), *Oas2* (dMmuCPE5099434, BioRad), *Axl* (dMmuCPE5090992, BioRad), *Eif4h* (forward: 5′ TGCAGCTTGCTTGGTAGC, reverse: 5′ GTAAATTGCCGAGACCTTGC, probe: 5′HEX-AGCCTACCCCTTGGCTCGGG), *Rpp30* (forward: 5′TGACCCTATCAGAGGACTGC, reverse: 5′ CTCTGCAATTTGTGGACACG, probe: 5′HEX- TGGGCTTTCTGAAAATGATGGCAA) and *C4b* (forward: 5′ AGCCTGTTTCCAGCTCAAAG, reverse: 5′ GTCCTAAGGCCTCAC ACCTG, probe: 5′FAM- CCCCGGCTGCTGAACTCCAT).

### Microglia bulk RNAseq and analysis

One thousand and two hundred microglia from anterior cortex, spanning the frontal, motor and part of the somatosensory cortices were sorted into TCL buffer supplemented with 1% β-mercaptoethanol, and RNA was extracted using RNAclean XP beads as described. cDNA was synthesized using the SMART-Seq v4 ultra-low input kit for RNA sequencing (Takara Bio) according to manufacturer instructions. Library preparation for sequencing was performed using the Nextera XT DNA library prep kit (Illumina) according to the manufacturer’s instructions. Sample quality was assayed as recommended using the high sensitivity DNA kit (Agilent). Samples were pooled equimolarly and sequencing was performed on a Nextseq 500 sequencer (Illumina) with 1 nM input. For bulk RNAseq analysis, 75 bp single-end reads were first trimmed of their adaptors and then aligned to the mm10 mouse reference genome using STAR Aligner [29]. All samples had at least 20 million uniquely mapped reads. The raw read count matrix was then analysed in the EdgeR package [30] for differential gene expression using the QLF model. Gene ontology analysis was performed using the gProfiler2 package [31].

### Statistical analysis

For all statistical analysis, R and GraphPad Prism 8 (GraphPad Software) were used. Error bars represent s.d. in all figures. All replicate numbers, statistical tests, *p* values and *q* values used are indicated in the figure legends where appropriate. Statistical tests were selected based on the normality of the data distribution, assessed by the Shapiro-Wilk test or quantile-quantile (QQ) plot, and also the equality of variance, assessed by the *F* test.

## Results

### Titration of peripherally administered mIFNα

**Fig. 1:**
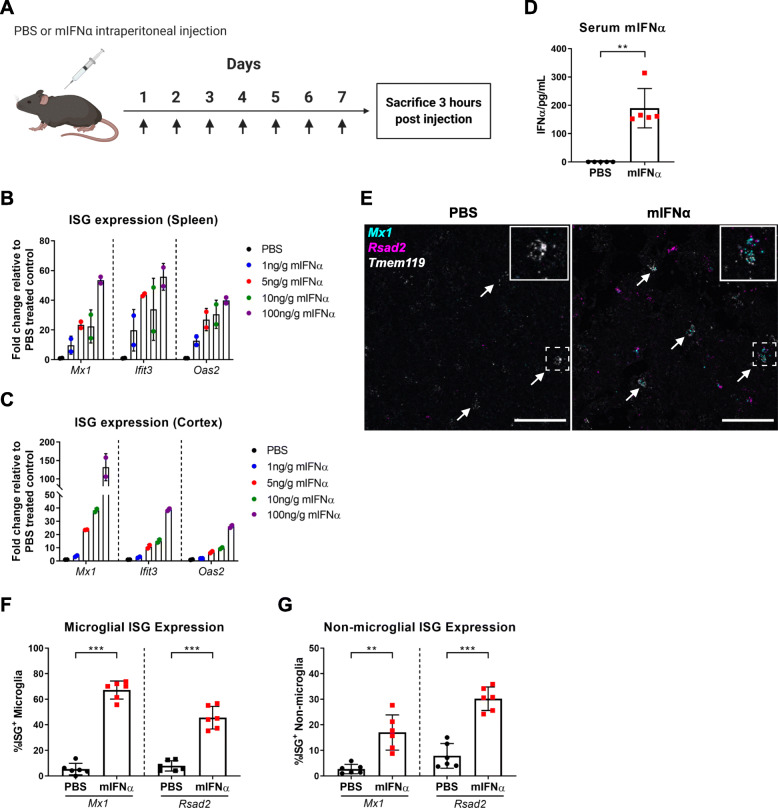
Induction of IFNAR signalling within the brain parenchyma with peripheral mIFNα administration. **a** Schematic of treatment protocol. Mice (*N* = 2) were injected daily with varying doses of mIFNα for 7 days and sacrificed 3 h post final injection. Tissue samples were harvested following perfusion with ice-cold PBS to remove contamination from peripheral blood cells. **b** Interferon-stimulated gene (ISG) expression was assayed in the spleen using ddPCR, showing a dose-dependent effect of mIFNα on ISG expression. **c** ISG expression was similarly assayed in the cortex, showing a dose-dependent effect on ISG expression. **d** Serum mIFNα from PBS or mIFNα injected mice (*N* = 5 per group) was assayed using ELISA. Serum was collected 3 h post final injection. **e** Representative RNAscope images showing enriched ISG (*Mx1*, *Rsad2*) transcript abundance in *Tmem119*-positive microglia (white arrows) in mIFNα-treated mice. Inset in each image shows an example of ISG negative or positive microglia. **f**, **g** Quantitative analysis of RNAscope data showing significant ISG enrichment in both microglia (*Tmem119* positive) and non-microglia (*Tmem119* negative) cell types with mIFNα exposure (*N* = 6 per condition). Scale bar = 50 μm. **p < 0.*01*; ***p < 0.*001*; Student’s *t* test or Mann-Whitney *U* test, assessed by normality of data distribution

In order to determine whether peripherally administered murine recombinant interferon α (mIFNα) was able to induce a signalling effect within the central nervous system (CNS), mice were injected intraperitoneally with varying doses of mIFNα. To model acute type 1 interferon-mediated inflammation, mice were injected daily for 7 days (Fig. 1a), and a dose-dependent, titratable effect on interferon-stimulated gene (ISG) expression both in the spleen (Fig. 1b) and in perfused cortical tissue from the brain (Fig. 1c) was observed. A working dose of 10 ng mIFNα/g of body weight was selected and used for all further experiments, which is of similar dosage used in previous studies and also within the relative dose range that has been used therapeutically in patients with chronic hepatitis C infection or various cancers [19, 32–36]. Furthermore, serum concentrations of mIFNα averaged at around 200 pg/mL 3 h post final injection using this dosing schedule (Fig. 1d), indicating a roughly 600-fold decrease in bioavailability at this time point, assuming a total blood volume of 2 mL for a 25-g male mouse. Given the induced ISG signature, we observed in the spleen and brain cortex at this same time point, it is reasonable to assume that at least part of this large decrease in bioavailability was due to cellular or tissue consumption and signal transduction of mIFNα as manifested by their ISG signature.

### Peripherally administered mIFNα induces ISG expression in microglia

The blood-brain barrier (BBB) tightly regulates the movement of a wide range of molecules, including cytokines, to and from the CNS [37]. Given the recent appreciation that microglia play critical immunological roles in surveying and protecting the CNS from pathogenic threats and injury [12, 13, 25], it was next investigated whether peripherally administered mIFNα would be able to induce IFNAR signalling in microglia across the BBB. Indeed, using RNAscope in situ hybridization, peripherally administered mIFNα increased expression of the ISGs *Mx1* and *Rsad2*, in up to 75% of microglia (*Tmem119*-positive cells) within the frontal cortex (Fig. 1e, f). We note that increased numbers of ISG puncta were also present in non-*Tmem119* positive cells (Fig. 1g), suggesting that peripherally administered mIFNα is also able to induce functional IFNAR signalling in cell types other than microglia within the CNS.

### A genetic and phenotypic mIFNα signature in microglia

**Fig. 2:**
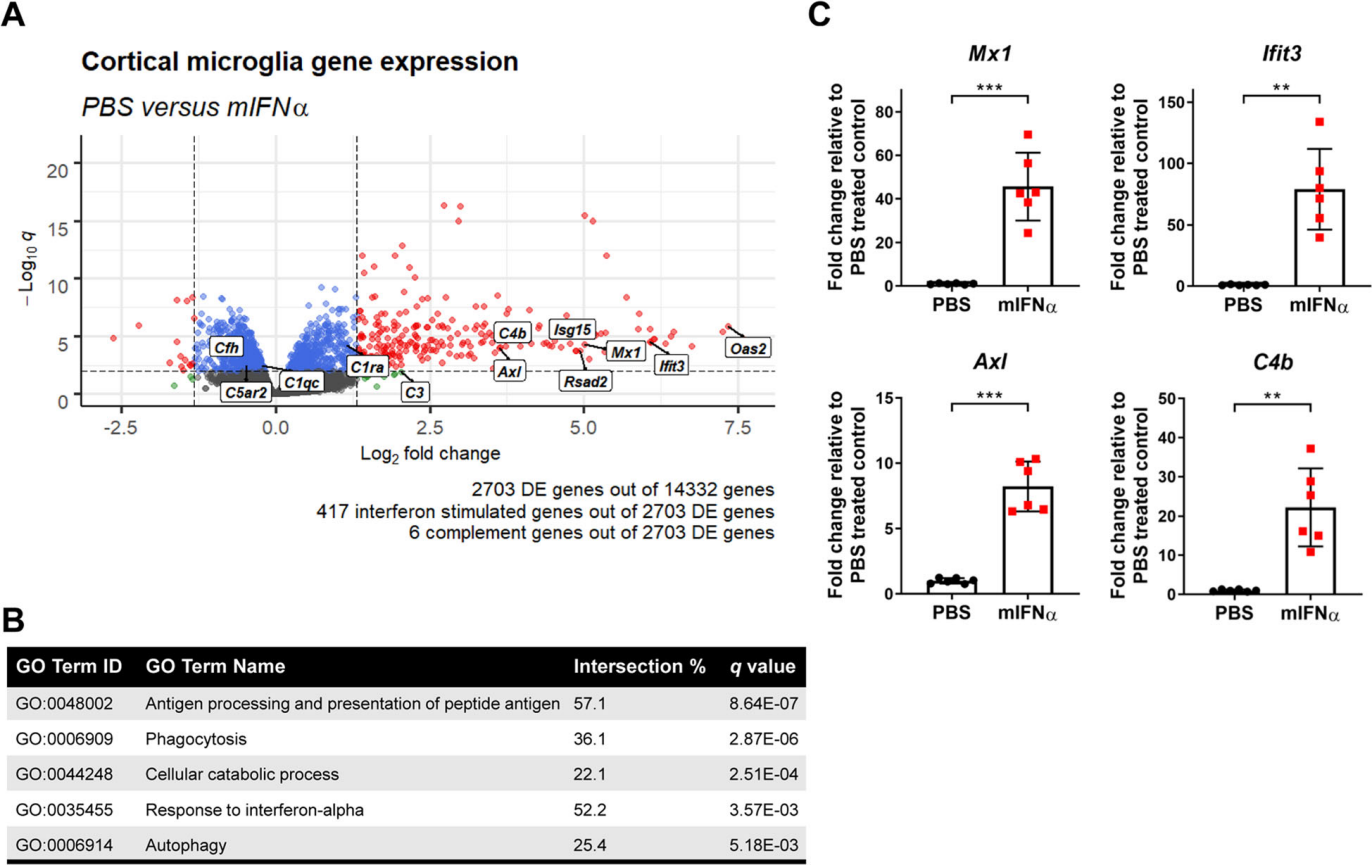
Transcriptional profiling of mIFNα exposed microglia. **a** Volcano plot showing highlights of differentially expressed (DE) genes from bulk RNA sequencing of sorted cortical microglia. Interferon-stimulated genes were identified based on querying the Interferome 2.0 database [43] with the DE gene list. *q*, Benjamini-Hochberg adjusted *p* value. Dotted lines demarcate *q* = 0.01 and fold change = 2.5. **b** A selection of differential gene ontology pathways identified using gProfiler2 [31] enriched in mIFNα exposed microglia. **c** Validation of several interferon-stimulated genes (ISGs) by ddPCR on sorted cortical microglia from an independently treated cohort of mice. *N* = 6 for all data shown. **p < 0.*01*; ***p < 0.*001*; Student’s *t* test

**Fig. 3:**
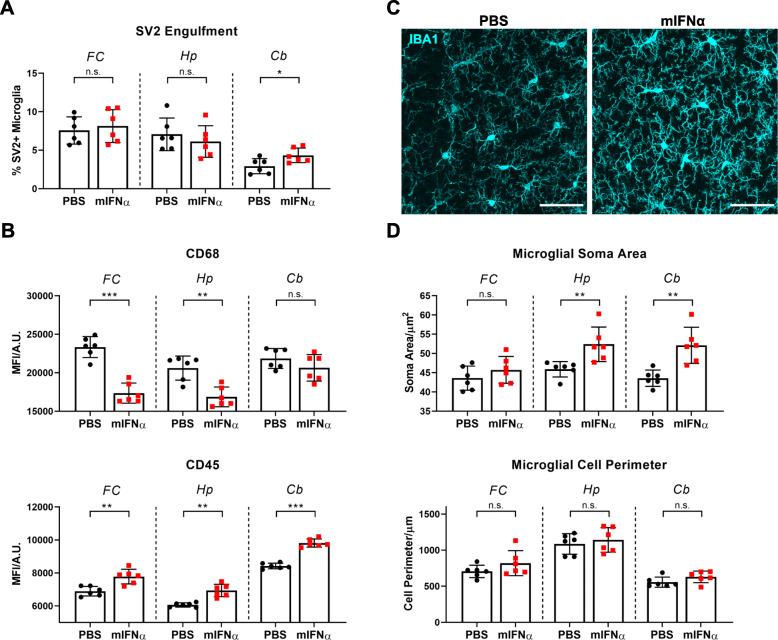
Microglial IFNAR signalling results in unique changes in classical activation parameters. **a** Selectively enhanced microglial synapse engulfment in the cerebellum with mIFNα exposure. **b** mIFNα-induced changes in microglial surface expression of CD68 (decrease) and CD45 (increase) across different brain regions. **c** Representative images of microglia from the hippocampus from both treatment groups. Scale bar = 50 μm. **d** Analysis of morphological changes in microglia with mIFNα exposure, showing brain region-specific differences. *N* = 6 for all data shown. n.s., *p > 0*.*05*; *p < 0.*05*; **p < 0.*01*; ***p < 0.*001*; Student’s *t* test or Mann-Whitney *U* test, assessed by normality of data distribution

IFNα therapy commonly induces a variety of sickness behaviours that include fatigue, anhedonia and paresthesia [5, 38, 39]. While the neurological bases of these sickness behaviours remain incompletely characterized, they have been associated with specific brain regions including the prefrontal and sensorimotor cortices [40–42]. Therefore, in order to further investigate mIFNα-induced genetic changes in microglia in a behaviourally relevant brain region, bulk RNA sequencing on sorted microglia from the anterior cortex spanning the frontal, motor and part of the somatosensory cortices (Supplementary Figure 1) was performed. Analysis of differentially expressed (DE) genes revealed the expected upregulation of many ISGs, as well as a few members of the complement gene family, in particular *C4b* (Fig. 2a). Additionally, mIFNα treatment also altered the expression profile of several microglial cytokine and sensome [44] genes (Supplementary Figures 1D, E), the latter being previously defined as a set of protein encoding genes that function as sensors of endogenous ligands and microbes. We next performed gene ontology (GO) analysis using gProfiler2 [31] to identify functional pathways altered by mIFNα (Fig. 2b). As expected, type 1 IFN response pathways, which included the phagocytic and class 1 MHC antigen presentation programmes, were found to be significantly altered. Many significantly altered GO terms relating to cellular metabolism, which included the terms “autophagy” and “cellular catabolic process”, were also observed. Finally, the increased expression of some of the identified DE genes was validated by ddPCR of sorted cortical microglia from mIFNα-treated mice (Fig. 2c). Aberrant synapse pruning by microglia has been implicated in both neurodegenerative and neuropsychiatric disease [14, 17], and it remains unclear whether acute exposure to mIFNα would result in any changes to microglia-mediated synapse pruning. It has been previously shown that peripheral IFNα injection induces an enriched ISG signature in the hippocampus and cerebellum compared to other anatomical regions in the rodent brain [33]. Given the known associations of these brain regions to the cognitive deficiencies and anxiety behaviours associated with IFNα treatment [45–48], microglia-mediated synaptic engulfment was assessed in the hippocampus and cerebellum in addition to the frontal cortex, which has also been implicated in these sickness behaviours [46, 49]. Using a flow cytometry-based method to detect intracellular presynaptic particles (SV2) within microglia (Supplementary Figure 2), it was found that peripheral mIFNα treatment only modestly increased intracellular SV2 in cerebellar microglia, but not in microglia from either the frontal cortex or hippocampus (Fig. 3a). To ascertain if there were any changes in expression of markers of microglial activation, flow cytometric analyses of CD68 and CD45 expression levels, which are associated with microglial activation and inhibition of activation, respectively [50, 51], were performed. There was a statistically significant decrease in CD68 expression in microglia from both the frontal cortex and hippocampus, and a trend towards decreased expression in the cerebellum (Fig. 3b). CD45 expression was significantly increased in all 3 brain regions assayed (Fig. 3b), suggesting a classically defined hypo-activated microglial state. Furthermore, immunohistological analyses of microglial morphology suggested a unique mIFNα-induced morphological state, with microglia displaying significantly increased soma size (associated with microglial activation), and a trend towards increased cell perimeter, which was used as a proxy measure for process ramification (Fig. 3c, d). These results suggest the possibility that acute mIFNα exposure induces a unique phenotypic state within microglia that displays subtle brain region-specific variability, which could potentially be due to region-specific levels of immune signalling module expression in microglia [52].

### Knockdown of microglial IFNAR signalling attenuates the mIFNα genetic signature and phenotype

Due to the ubiquitous nature of IFNAR expression in multiple cell types, it is possible that mIFNα might induce secondary signalling effects on microglia that are downstream of IFNAR signalling in these different cell types. For example, these effects could occur via the production of other cytokines or chemokines, such as CXCL10 from brain endothelial cells [53], or even IL-6, IL-1β and TNFα from microglia [19] in response to type 1 interferon signalling. Indeed, significant gene expression upregulation of several of these cytokines and chemokines in mIFNα-exposed microglia were observed (Supplementary Figure 1E), which could potentially contribute towards autocrine signalling effects. Furthermore, many ISGs can also be cross regulated by distinct signalling pathways downstream of other cytokines, such as IL-1β, IFNγ and TNFα [54–57]. Given the increased expression of some of these cytokines in response to IFNα (Supplementary Figure 1E), it is possible that the observed canonical ISG signature is additionally derived in part by other cytokine signalling pathways.

**Fig. 4:**
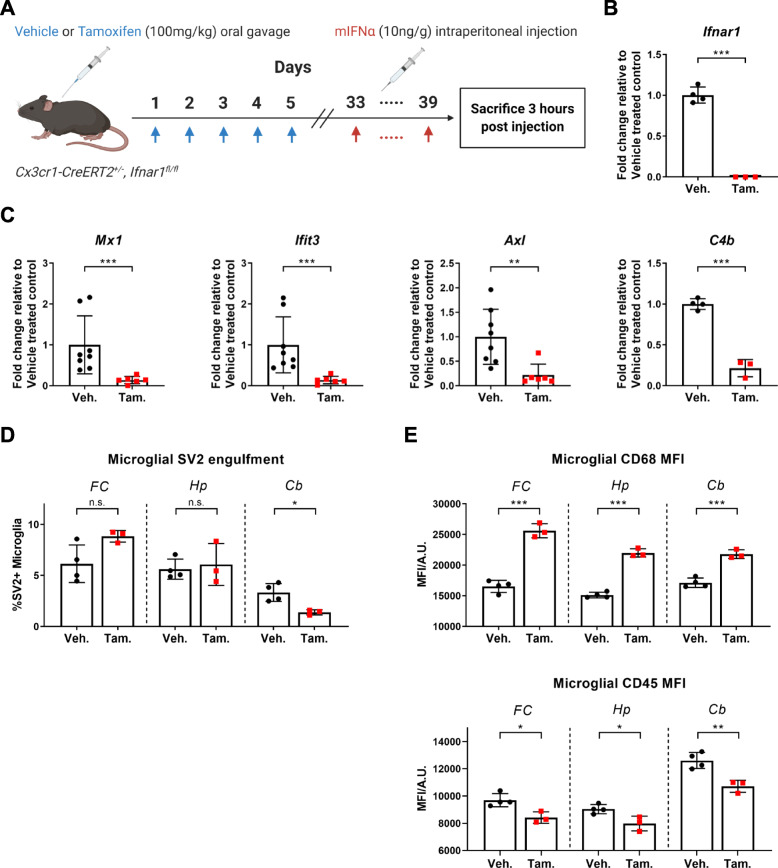
Knockdown of microglial IFNAR signalling attenuates phenotypic and genetic changes in response to peripheral mIFNα administration. **a** Schematic of treatment protocol. *Cx3cr1*-*CreERT2*^*+/−*^, *Ifnar1*^*fl/fl*^ mice were orally gavaged with either vehicle or tamoxifen. Four weeks post gavage, mice were either sacrificed (for qPCR validation of *Ifnar1* knockdown) or treated with mIFNα daily for 7 days and sacrificed. **b** Cortical microglia from *Cx3cr1*-*CreERT2*^*+/−*^, *Ifnar1*^*fl/fl*^ mice were sorted by FACS and analysed for gene expression of *Ifnar1* by qPCR. *Ifnar1* qPCR primers were designed to span the floxed region of Exon 10. The tamoxifen-treated (*N* = 3) CreERT2-positive group showed significant knockdown of *Ifnar1* expression compared to the vehicle-treated group (*N* = 4). **c** Sorted cortical microglia show significantly decreased ISG expression by ddPCR in the tamoxifen treatment group (*N* = 6) of *Cx3cr1*-*CreERT2*^*+/−*^, *Ifnar*^*fl/fl*^ mice in comparison to the vehicle treatment group (*N* = 8). *C4b* expression was also significantly decreased in the tamoxifen treatment group (*N* = 3) in comparison to the vehicle treatment group (*N* = 4). **d** Knockdown of microglial *Ifnar1* in the tamoxifen treatment group (*N* = 3) reverses the observed region-specific changes in synapse engulfment induced by peripheral mIFNα administration in comparison to the vehicle treatment group (*N* = 4). **e** Knockdown of microglial *Ifnar1* in the tamoxifen treatment group (*N* = 3) reverses the observed changes in CD45 and CD68 expression induced by peripheral mIFNα administration, with the exception of CD68 expression in the cerebellum in comparison to the vehicle treatment group (*N* = 4). Veh., Vehicle; Tam., Tamoxifen; n.s., *p > 0*.*05*; *p < 0.*05*; **p < 0.*01*; ***p < 0.*001*; Student’s *t* test or Mann-Whitney *U* test, assessed by normality of data distribution

In order to further investigate whether the IFNα-induced phenotypic and genetic changes in microglia can primarily be attributed to direct microglial IFNAR signalling, *Ifnar1* expression on microglia was selectively knocked down using the inducible *Cx3cr1*-*CreERT2* driver [26] (Supplementary Figure 3; Fig. 4a, b). Indeed, mIFNα treatment of mice with microglial specific knockdown of *Ifnar1* resulted in the attenuation of ISG expression, as well as *C4b* expression, in microglia compared to control mice (Fig. 4c), suggesting that these genes are directly regulated by microglial IFNAR signalling. Furthermore, there was a reversal of the SV2 engulfment phenotype in cerebellar microglia with knockdown of microglial *Ifnar1*, with no corresponding significant change in microglial SV2 engulfment in both the frontal cortex and hippocampus (Fig. 4d). Changes in microglial CD68 and CD45 expression levels were also largely reversed with knockdown of microglial *Ifnar1* (Fig, 4e).

**Fig. 5:**
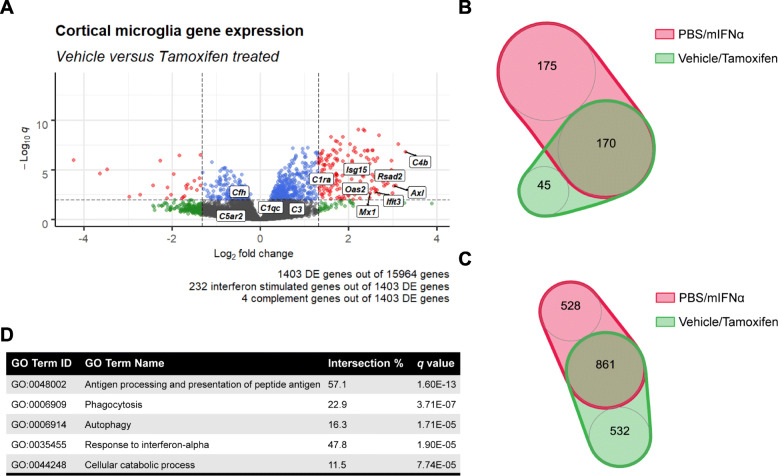
Transcriptional profiling of *Ifnar1* sufficient and deficient microglia exposed to mIFNα. **a** Volcano plot showing highlights of differentially expressed (DE) genes from bulk RNA sequencing of sorted cortical microglia, comparing vehicle-treated control (*N* = 6) and tamoxifen-treated knockdown (*N* = 6) groups from *Cx3cr1*-*CreERT2*^*+/−*^, *Ifnar1*^*fl/fl*^ mice. Interferon-stimulated genes were identified based on querying the Interferome 2.0 database [43] with the DE gene list. *q*, Benjamini-Hochberg adjusted *p* value. **b** Overlap of independently identified > 1.5-fold change DE genes from both sequencing libraries, identified using the glmTreat function in the EdgeR package [58]. Venn diagram sizes are proportional to the numerical values in each category. **c** Overlap of independently identified gene ontology pathways. Venn diagram sizes are proportional to the numerical values in each category. **d** A selection of differential gene ontology pathways identified using gProfiler2 [31] enriched in mIFNα exposed *Ifnar1* sufficient and deficient microglia

Finally, the possibility of any potential non-microglial IFNAR signalling effects was investigated by bulk RNA sequencing of microglia sorted from the same anterior cortex region of *Ifnar1* sufficient and deficient mice treated with mIFNα (Supplementary Figure 4). As expected, knockdown of *Ifnar1* on microglia attenuated many of the genes induced by mIFNα as described earlier (Fig. 5a), with most of the DE genes displaying the highest log_2_ fold change (> 1.5) overlapping in identity with similarly high log_2_ fold change DE genes from mIFNα-exposed wild type microglia (Fig. 5b). Furthermore, the GO terms from both sets of experiments remained similar (Fig. 5c, d). Finally, the panel of sensome and cytokine/chemokine genes that were differentially expressed remained similar as well (Supplementary Figures 4C, D).

These data suggest that peripherally derived IFNα can induce a functional signalling event through microglia by upregulating a unique genetic and phenotypic programme. These genetic and phenotypic changes rely primarily on microglial IFNAR signalling and are largely independent of any potential secondary signalling effects through other cytokines or chemokines.

## Discussion

To investigate the interaction of peripheral IFNα with the CNS, in particular microglia, we first show that peripherally administered mIFNα is able to exert direct functional signalling effects across the BBB on microglia. This is in agreement with previous studies which have shown that IFNα can enter the brain parenchyma from the periphery via a non-saturable mechanism [59, 60], and also elicit a transcriptional response within numerous cell types in the brain parenchyma [33]. Gene expression differences were further characterized by RNA sequencing and showed an expected strong upregulation of genes associated with type 1 interferon signalling. Interestingly, we also found a strong increase in expression of the complement component *C4b*, which has previously been observed in the contexts of viral infection [61] and ageing [21, 23], in microglia that also concurrently express a strong type 1 interferon signature. In particular, we also show that IFNAR signalling on microglia results in a unique phenotype characterized by subtle morphological changes and changes in the classical activation markers CD45 and CD68 in a region-specific manner. Furthermore, we also show an increase in microglial synaptic engulfment within the cerebellum but not in the hippocampus or frontal cortex with mIFNα exposure.

By specifically knocking down *Ifnar1* expression on microglia through the use of the inducible *Cx3cr1*-*CreERT2* driver, we show that these genetic and phenotypic changes are largely due to direct signalling through microglial IFNAR. We acknowledge that direct comparison between our RNAseq datasets is not possible due to the experimental design and our resulting inability to correct the data for batch effects. We also cannot rule out the likely possibility that incomplete knockdown of *Ifnar1* on microglia diluted the significance and magnitude of DE genes, and consequently, gene ontology pathways identified. Nevertheless, we show that a sizeable proportion of highly DE genes and gene ontology pathways overlap across both datasets, suggesting a major contribution of direct microglial IFNAR signalling despite the experimental caveats.

It is increasingly appreciated that the complement pathway might play a significant role in the pathogenesis of several neurodegenerative [14] and neuropsychiatric [16] diseases and that it is also a driver of synapse loss in viral infection-associated cognitive decline [25]. In particular, recent studies have shown that overexpression of mouse C4 within the mouse prefrontal cortex or human C4A in the whole brain is sufficient to induce increased microglial synaptic pruning that results in impaired social behaviour [62]. We observed a modest, but statistically significant, increase in microglial presynaptic engulfment in the cerebellum, but not frontal cortex and hippocampus. While our RNA sequencing and ddPCR validation studies showed an mIFNα-induced increase in *C4b* expression on sorted anterior cortex microglia, it is likely that cerebellar microglia also increase *C4b* expression in response to mIFNα, given that at baseline they are in a more “immunologically vigilant” state with an enriched type 1 IFN regulatory signature, compared to cortical and hippocampal microglia [52]. The increase in presynaptic engulfment by cerebellar microglia compared to microglia from the other brain regions could thus be due to an enhanced signalling response to mIFNα, a facet of which could include higher *C4b* expression. Additionally, it is probable that within an acute period of mIFNα exposure, most neurons or synapses still express sufficient signals to prohibit synaptic engulfment, such as CD47 [63] and other complement regulatory factors. These regulatory signals and factors could be differentially distributed throughout different regions of the murine brain, resulting in differing levels of inhibition of complement activation and deposition. It should also be noted that our flow cytometric assay for synapse engulfment does not take into account any potential differences in the process of cargo degradation within the phagocytic pathway, although no significant differences in SV2 signal were observed when wild type, cortical microglia were exposed to chloroquine, an inhibitor of lysosomal acidification and acid-sensitive lysosomal proteases (data not shown). Furthermore, a previous study did not observe any differences in sensorimotor behaviour of mice injected daily with mIFNα for up to 5 weeks at a similar dose [32], which suggests that the small increase in synaptic engulfment we observe in the cerebellum might be phenotypically insignificant. However, it is possible that chronic IFNAR signalling within microglia and other CNS cell types could result in elevated C4 production and decreased complement and phagocytic inhibitory factors that over time could lead to the pathologic pruning of synapses. Of note, it was shown in the same study that chronic IFNα exposure (~ 4–5 weeks) in mice adversely affected neurogenesis and resulted in depressive behaviour [32]. Notably, endogenous IFNα can biochemically, and presumably functionally, cross signal through μ-opioid receptors to exert anti-nociceptive effects [64, 65]. It would therefore also be possible that the pathological effects of IFNα are mediated in part, or in tandem, through these secondary, or non-IFNAR signalling related processes.

Importantly, our study also shows a unique IFNα-induced gene signature in microglia that bears similarity to other datasets presenting an enriched type 1 interferon signature in microglia within several pathological and biological contexts. GO analysis identified the expected terms for interferon responses and, also interestingly, metabolic changes in catabolism. While caution should be borne in interpreting the directionality of biological pathways based solely on gene ontology terms, we note that IFNα and viral infection have been found to generally induce a catabolic state in various cell types including macrophages [66–68]. Deficiencies in microglial autophagy have also been recently linked to impaired synaptic pruning and autism-like behavioural defects [17]. It would therefore be interesting to further probe how IFNAR signalling in microglia might modulate autophagic flux and whether increased autophagy in microglia might be a contributing mechanism for increased synaptic pruning in the context of chronic type 1 interferon exposure during autoimmune disease such as SLE.

## Conclusion

In summary, we show that peripherally derived IFNα is able to signal directly across the BBB through microglial IFNAR. This results in a unique genetic and phenotypic profile that is primarily dependent on IFNAR signalling with minimal contribution from other secondary cytokine or chemokine signalling pathways. IFNα-induced upregulation of *C4b* and other ISGs might regulate various cellular functions and processes such as synaptic pruning in a brain region-dependent manner. These findings would be of further investigative interest given the long-standing association of endogenous and therapeutic IFNα with neuropsychiatric symptoms and the emerging therapeutic interest in IFNα use for prophylactic treatment of COVID19 [69–71].

## Supplementary Information

**Additional file 1: Figure S1.** Microglia sorting and RNAseq data analysis. (A) Gating strategy for microglia cell sorting. (B) Principle component analysis (PCA) plot showing unbiased clustering of samples based on treatment group (N = 6 per group). PC, Principal component. (C) Microglia specific genes were highly expressed in sorted microglia from both treatment groups. (D) Expression profile showing Log_2_ fold change of microglial sensome genes [44] that are differentially expressed with mIFNα treatment. (E) mIFNα treatment induces significant changes in microglial gene expression of cytokines and chemokines. Normalized counts and differential expression testing were generated and performed using the EdgeR package [30].

**Additional file 2: Figure S2.** Flow cytometric assay of microglial synapse engulfment. (A) Schematic of experimental procedure for flow cytometric assaying of microglial SV2+ presynaptic engulfment. 10 μM Cytochalasin D was added in buffers used during dounce homogenization and Percoll gradient centrifugation. (B) Effect of cytochalasin D treatment on the SV2 engulfment assay, showing the inhibition of active microglial phagocytosis of SV2 particles during tissue processing. Cortical tissue from opposing hemispheres in each mouse was perfused, dissected and processed with or without cytochalasin D, allowing for paired analysis of phagocytosis inhibition within the same mouse (N = 9). Veh., Vehicle; Cyto. D, Cytochalasin D; **, *p < 0*.*01*; Wilcoxon’s signed rank test. (C) Flow cytometric gating strategy for the engulfment assay. (D) Lack of neuronal debris adhering to microglia. Non permeabilized microglia show no fluorochrome signal compared to permeabilized microglia (N = 3 per treatment). P, Permeabilized; NP, Non-permeabilized; *, *p < 0*.*05*; Student’s t test. (E) Isotype controls showing absence of non-epitope driven binding of SV2 and CD68 antibodies during intracellular staining.

**Additional file 3: Figure S3.** Validation of *Cx3cr1*-*CreERT2* targeting of microglia. (A) Schematic of treatment protocol. *Cx3cr1*-*CreERT2*^*+/−*^, *R26*-*EYFP*^*LSL*^ mice were orally gavaged with either vehicle (N = 2) or tamoxifen (N = 3) and sacrificed 4 weeks post gavage. (B) Representative confocal images of frontal cortex of *Cx3cr1*-*CreERT2*^*+/−*^, *R26*-*EYFP*^*LSL*^ mice showing specific expression of the *Cx3cr1*-*CreERT2* transgene in IBA1 expressing microglia/myeloid lineage cells. Scale bars = 200 μm. (C) Flow cytometric analysis of YFP expression in CD11b^+^, CD45^lo^ microglia, showing elevated YFP expression only in the tamoxifen treated group. Veh., Vehicle; Tam., Tamoxifen.

**Additional file 4: Figure S4.** RNAseq analysis of anterior cortex microglia sorted from *Ifnar1* sufficient and deficient mice treated with mIFNα. (A) Principle component analysis (PCA) plot showing unbiased clustering of samples based on treatment group (N = 6 per group). PC, Principal component. (B) Microglia specific genes were highly expressed in sorted microglia from both treatment groups. (C) Expression profile showing Log_2_ fold change of microglial sensome genes [44] that are significantly differentially expressed in *Ifnar1* sufficient vs. deficient microglia. (D) mIFNα treatment induces changes in microglial gene expression of cytokines and chemokines. Normalized counts and differential expression testing were generated and performed using the EdgeR package [30].

## Data Availability

All datasets used and/or analysed during the current study are available from the corresponding author on reasonable request. Bulk RNA sequencing data may be accessed at the NCBI SRA with accession ID PRJNA658781.

## Abbreviations

AGS: Aicardi-Goutières syndrome
BBB: Blood-brain barrier
CNS: Central nervous system
DE: Differentially expressed
GO: Gene ontology
IFNα: Interferon alpha
IFNγ: Interferon gamma
mIFNα: Murine interferon alpha
IFNAR: Type 1 interferon receptor
IL-1β: Interleukin 1 beta
IL-6: Interleukin 6
IRF9: Interferon regulatory factor 9
ISG: Interferon-stimulated gene
ISGF3: Interferon-stimulated gene factor 3
ISRE: Interferon-stimulated response element
JAK1: Janus kinase 1
RTK: Receptor tyrosine kinase
SLE: Systemic lupus erythematosus
STAT1: Signal transducer and activator of transcription 1
STAT2: Signal transducer and activator of transcription 2
SV2: Synaptic vesicle 2
TNFα: Tumour necrosis factor alpha
TYK2: Tyrosine kinase 2
