# Supplementary material for: Microglial responses to peripheral type 1 interferon
Source: J Neuroinflammation. 2020 Nov 12;17:340. doi: 10.1186/s12974-020-02003-z (PMC7659169; doi:10.1186/s12974-020-02003-z)

Supplementary Figure 3

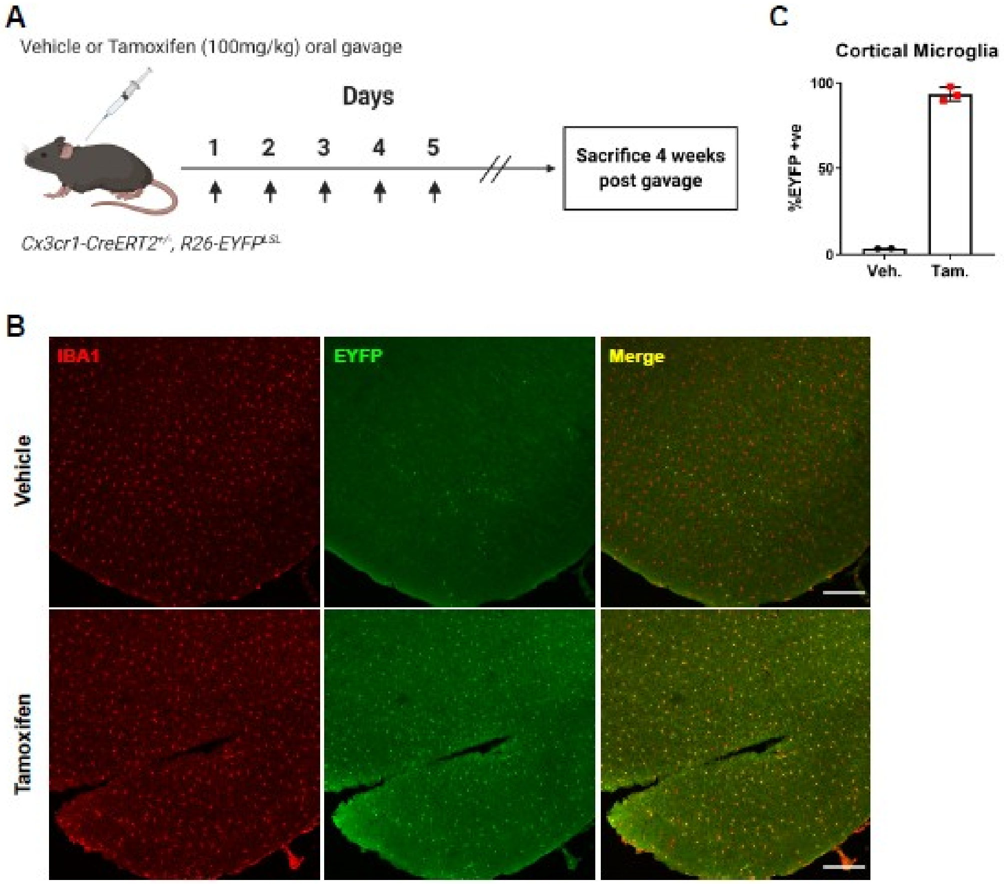

Supplement: Supplementary file 3 [file 12974_2020_2003_MOESM3_ESM.docx]
